# Supplementary material for: Artificial Intelligence Applied to Colonoscopy: Is It Time to Take a Step Forward?
Source: Cancers (Basel). 2023 Apr 7;15(8):2193. doi: 10.3390/cancers15082193 (PMC10136753; doi:10.3390/cancers15082193)
Supplement: Supplementary file 1 [file cancers-15-02193-s001.zip › cancers-2290111-supplementary.pdf]

Supplementary Materials: Artificial Intelligence Applied to Colonoscopy: Is it Time to Take a Step Forward?

Antonio Z. Gimeno-García, Anjara Hernández-Pérez, David Nicolás-Pérez and Manuel Hernández-Guerra

Table S1. Computer-assisted diagnosis (CADx) studies (adapted and modified from references [1-3]).

Author, year (reference)	Design	Adquisition of information	Training / testing sets	Type of AI	Endoscopic image technology	S/E/PPV/NPV/ AC	Postpolypectomy surveillance interval agreement
Takemura, 2010[4]	R	Still images	Training set: 72 polyps. Testing set: 134 images.	Automatic quantification and classification of pit patterns	Magnification chromoendoscopy	S: 97.8% E: 97.8% PPV and NPV no reported AC: 98.5%	Not studied
Tischendorf, 2010[5]	P	Still images	Training set: 209 polyps. Testing set: not specified.	Vascularization features. Automated classification with SVM.	NBI + magnification colonoscopy	S: 90 % E: 70 % PPV and NPV no reported AC: 85.3 %	Not studied
Gross, 2011[6]	P	Still images	Training set: 434 polyps. Testing set: not specified.	Differentiation between neoplastic and non-neoplastic colorectal polyps. Automated classification with SVM.	NBI + magnification colonoscopy	S: 95.0 % E: 90.3 % PPV: 93.5 % NPV: 92.4 % AC: 93.1 %	Not studied
Takemura, 2012[7]	R	Still images	Training set: 1519 polyps. Testing set: 371 polyps	Predicting the histology of colorectal tumors on narrow-band imaging magnifying colonoscopy images. Automated classification with SVM.	NBI + magnification colonoscopy	S: 97.8 % E: 97.9 % PPV: not reported. NPV: not reported AC: 97.8 %	Not studied
Aihara, 2013[8]	P	Diagnosis in real-time	32 patients with 102 colorectal lesions (no training / testing sets)	Software that facilitates real-time numerical color analysis of still images	Autofluorescence endoscopy	S: 94.2 % E: 88.9 % PPV: 95.6 % NPV: 85.2 % AC: not reported	Not studied

Mori, 2015[9]	R (pilot study)	Still images	Testing set: 176 polyps. No training set	Fully automated diagnostic system (EC-CAD) for nuclear segmentation and classification	Endocytoscopy in polyps < 10 mm	S: 92 % E: 79 % PPV: not reported NPV: not reported AC: 89.2 %	Not studied
Mori, 2016[10]	R	Still images	Training set: 6051 images. Testing set: 205 polyps.	Fully automated diagnostic system (EC-CAD) for nuclear segmentation and classification. Automated classification with SVM.	Endocytoscopy in polyps < 10 mm	S: 88 % E: 92 % PPV: 99 % NPV: 85 % (97 % for rectosigmoid polyps) AC: 89 %	96 – 98 %
Kominami, 2016[11]	P	Diagnosis in real-time	Training set: 2247 images. Testing set: 118 polyps.	Prediction of histologic diagnoses of colorectal lesions. Automated classification with SVM.	NBI + magnification colonoscopy	S: 93 % E: 93.3 % PPV: 93 % NPV: 93.3 % AC: 93.2 %	92.7 %
Misawa, 2016[12]	P	Still images	Training set: 979 images. Testing set: 100 images.	Prediction of histologic diagnoses. Automated classification with SVM.	Narrow-Band Imaging Endocytoscopy	S: 84.5 % E: 97.6 % PPV: 98 % NPV: 82 % AC: 90 %	Not studied
Komeda, 2017[13]	R	Still images from videos	Training set: 1800 images. Testing set: 10 images.	Distinction between adenomatous or nonadenomato us lesion with CNN.	white-light colonoscopy, narrow-band imaging and chromoendosc opy.	AC: 70%.	Not studied.
Chen, 2018[14]	P	Diagnosis in real-time	Training set: 2157 images. Testing set: 284 images.	Deep neural network in polyps < 5 mm.	NBI	S: 96.3 % E: 78.1 % PPV: 89.6 % NPV: 91.5 % AC: 90.1 %	Not studied
Mori, 2018[15]	P	Diagnosis in real-time	Training set: 2157 images. Testing set: 466 polyps.	Microvascular evaluation with NBI mode and cellular visualization	Endocytoscopy , NBI and methylene	S: 91.3 – 93.8 % E: 88.7 – 91 % PPV: 92.9 – 94.4 %	Not studied.

				after staining with methylene blue. Automated classification with SVM.	blue staining in polyps < 5 mm	NPV: 86.3 – 89.9 % (for rectosigmoid polyps: 93.7 – 96.5 %) AC: not reported.	
Byrne, 2019[16]	P	Diagnosis in real-time in videos.	Training set: 223 videos (validation set: 40 videos). Testing set: 125 videos.	Differentiation of adenomatous and hyperplastic diminutive colorectal polyps with CNN.	NBI	S: 98 % E: 83 % PPV: 90 % NPV: 97 % AC: 94 %	Not studied
Sanchez-Montes, 2019[17]	P	Still images.	Training set: not reported. Testing set: 225 polyps.	Histology prediction system based on colorectal polyp textural surface patterns (dysplastic vs nondysplastic polyps). Support vector machine	Colonoscopoy with high definition white light + magnification or chromoendoscopy + NBI.	S: 92.3 % E: 89.2 % PPV: 93.6 % NPV: 87.1 % (for rectosigmoid polyps < 5 mm: 96.7 %) AC: 91.1 %	Not studied
Jin, 2020[18]	P	Still images	Training set: 2150 polyps. Testing set: 300 polyps.	CNN for evaluation of diminutive colorectal polyps.	NBI	S: 81.6 % E: 90.8 % PPV: 90.8 % NPV: 76.7 % AC: 86.7 %	Not studied
Song, 2020[19]	R	Still images	Training set: 12480 images (624 polyps). Testing set: 545 polyps.	Classification of serrated lesiones, benign adenomas or superficial cancers and deep submucosal cancers with a deep learning model.	NBI + magnification	S: 58.8 – 84.1 % E: 75 – 93.7 % PPV: 47.6 - 78 % NPV: 67.7 - 95 % AC: 81.3 % (overall)	Not studied
Kudo, 2020[20]	R	Still images	Training set: 69142 images. Testing set: 100 images.	Analysis of cell nuclei, crypt structure, and microvessels in endoscopic images for distinguishing neoplasms from	Endocytoscopy + methylene blue staining or NBI	S: 96.9 % E: 94.3 - 100 % PPV: 96.9 - 100 % NPV: 94.3 – 94.6 % AC: 96 - 98 %	Not studied.

				non-neoplasms (polyps ≤ 10 mm).			
Zachariah, 2020[21]	P	Still images	Training set: 6223 Testing set: 634 images.	CNN for distinguishing adenomatous lesions vs hyperplastic / serrated lesions (any size).	White-light colonoscopy or NBI	S: 91 %<br>E: 88 %<br>PPV: 74 %<br>NPV: 97 %<br>AC: 89 %	94 %
Ozawa, 2020[22]	R	Still images	Training set: 16418 images (4752 polyps) and 4013 normal images) Testing set: 7077 images.	CNN for distinguishing adenomatous vs non-adenomatous lesions.	White-light colonoscopy or NBI	S: 92 %<br>E: not reported<br>PPV: 86 %<br>NPV: 85 - 90 %<br>AC: 83 %	Not studied
Zorron Cheng Tao Pu, 2020[23]	R	Still images	Training set: two sets of 1235 and 123500. Validation/testing set: 69 images.	CNN for lesiones detected by NBI (including serrated lesions).	White-light colonoscopy or NBI or BLI.	AC: 93 – 96%<br>S, E, PPV and NPV not reported.	Not studied
Zhou, 2020[24]	R	Still images	Training set: 5545 images. Testing set: 1451 images and 82 videos.	Deep learning Architecture for detection of serrated lesions and lateral spreading tumors.		S > 98%<br>E, PPV, NPV and AC not reported.	Not studied
van der Zanden, 2021[25]	P	Still images	Training set: 2449 images. Testing set: 60 images.	Artificial neural networks for classification of hyperplastic, adenomatous and serrated lesions.	High-definition white-light colonoscopy + BLI	S: 95.6 %<br>E: 93.3 %<br>PPV: 97.7 %<br>NPV: 87.5 %<br>AC: 86.7 – 95 %	Not studied
Weigt, 2022[26]	P	Still images and videos	Training set: 1202 polyps and 3571 frames without lesions. Testing set: 267 images.	CNN for classification of neoplastic vs non-neoplastic lesions.	White-light colonoscopy + BLI	S: 85 %<br>E: 78.9 %<br>PPV and NPV not reported.<br>AC: 83.6 %	Not studied
Hassan, 2022[27]	P	Diagnosis in real-time	Training set: 63,445 images (validation set: 8645 images;	CNN for differentiating between adenoma and	White-light colonoscopy without magnification	S: 82 %<br>E: 93.2 %<br>PPV: 65.3 %<br>NPV: 97.6 %	> 95 %

			pre-testing set: 26,412 images). Testing set: 295 lesions.	nonadenoma ≤ 5-mm rectosigmoid lesions.		AC: 91.8 %	
Hossain, 2023[28]	P	Still images	Training set: 55890 images (internal validation set: 8557 images). Testing set: 115 polyps.	Deep learning model for classification into adenomatous and non-adenomatous polyps.	White-light colonoscopy + NBI + BLI	S: > 92 %<br>E: 60 - 65 %<br>PPV: not reported<br>NPV: 75 - 86 %<br>AC: 82 - 85 %	> 90 %
Rondonotti, 2023[29]	P	Diagnosis in real-time	Same characteristics as described in Weigt *et al.* [26]	CNN (CAD_EYE) for characterization in BLI mode of rectosigmoid polyps (< 5 mm).	White-light colonoscopy BLI	S: 88.6 %<br>E: 88.1 %<br>PPV: 85.1 %<br>NPV: 91 %<br>AC: 88.4 %	> 92.6 %

AC: accuracy; AI: artificial intelligence; BLI: blue-laser imaging; CNN: convolutional neural network; E: specificity; NBI: narrow band imaging; NPV: negative predictive value; PPV: positive predictive value; P: prospective; R: retrospective; S: sensitivity; SVM: support vector machine.

Table S2. Artificial intelligence prediction of submucosal invasion in retrospective studies.

Author (year)	Study type	Aim	Number of patients	Sensitivity	Specificity	Accuracy	AUC
Minami et al. (2022)[30]	Retrospective Design and prospective validation of a CNN. Single center study	Differentiate shallow and deep submucosal invasion	196 patients :<br>- 91 Learning set (706 images)<br>- 49 validation set (394 images)<br>- 56 test set (560 images)	Validation set: 87.2%<br>Test set: 75.7%	Validation set: 35.7%	74.4%	0.758
Lui TKL et al (2019)[31]	Retrospective Design and prospective validation of a CNN. Single center study	Prediction of curative endoscopic resection	-Training set: 1692 lesions (8000 images)<br>- Test set: 76 lesions (567 images)	Test set: 88.2%	Test set: 77.9%	85.5%	0.837
Luo Y et al (2021)[32]	Retrospective Design and prospective validation of a CNN. Single center study	Differentiate shallow and deep submucosal invasion	813 lesions:<br>- 657 training set (7734)<br>- 156 test set (1634)	Test set (including advanced CRC): 91.2%<br>Test set (only early cancer): 65.3%	Test set(including advanced CRC): 91%´<br>Test set (only early cancer): 68.5%	91.1%(including advanced CRC):<br>Test set (only early cancer):68.3%	0.97(including advanced CRC):<br>Test set (only early cancer): 0.729
Tokunaga M et al (2021)[33]	Retrospective Design and prospective validation of a CNN. Single center study	Prediction of adequacy for curative endoscopic resection	1035 lesions:<br>- 824 training set (2751)<br>- 211 test set (691)	Test set (including advanced CRC): 96.7%	Test set(including advanced CRC): 75%´	Test set(including advanced CRC): 90,3%	Test set(including advanced CRC): 0.91%´

						Test set (only deep early cancer):51.2%	
Yao L et al (2022)[34]	Retrospective Design and validation of a CNN. Multicenter study	Differentiate shallow and deep submucosal invasion	533 lesions:<br>- 339 training set<br>- 194 test set	Test set (including advanced CRC): 78.8%<br>Test set (only early cancer): 50%	Test set (including advanced CRC): 96.2%<br>Test set (only early cancer): 96.2%	Test set (including advanced CRC): 90.4%<br>Test set (only early cancer): 91.2%	

AUC: area under the curve; CNN: convolutional neural network; CRC: colorectal cancer.

References

1. Pannala, R.; Krishnan, K.; Melson, J.; Parsi, M.A.; Schulman, A.R.; Sullivan, S.; Trikudanathan, G.; Trindade, A.J.; Watson, R.R.; Maple, J.T.; et al. Artificial intelligence in gastrointestinal endoscopy. *VideoGIE : an official video journal of the American Society for Gastrointestinal Endoscopy* **2020**, *5*, 598-613, doi:10.1016/j.vgie.2020.08.013.
2. Parsa, N.; Byrne, M.F. Artificial intelligence for identification and characterization of colonic polyps. *Therapeutic advances in gastrointestinal endoscopy* **2021**, *14*, 26317745211014698, doi:10.1177/26317745211014698.
3. Joseph, J.; LePage, E.M.; Cheney, C.P.; Pawa, R. Artificial intelligence in colonoscopy. *World journal of gastroenterology* **2021**, *27*, 4802-4817, doi:10.3748/wjg.v27.i29.4802.
4. Takemura, Y.; Yoshida, S.; Tanaka, S.; Onji, K.; Oka, S.; Tamaki, T.; Kaneda, K.; Yoshihara, M.; Chayama, K. Quantitative analysis and development of a computer-aided system for identification of regular pit patterns of colorectal lesions. *Gastrointestinal endoscopy* **2010**, *72*, 1047-1051, doi:10.1016/j.gie.2010.07.037.
5. Tischendorf, J.J.; Gross, S.; Winograd, R.; Hecker, H.; Auer, R.; Behrens, A.; Trautwein, C.; Aach, T.; Stehle, T. Computer-aided classification of colorectal polyps based on vascular patterns: a pilot study. *Endoscopy* **2010**, *42*, 203-207, doi:10.1055/s-0029-1243861.
6. Gross, S.; Trautwein, C.; Behrens, A.; Winograd, R.; Palm, S.; Lutz, H.H.; Schirin-Sokhan, R.; Hecker, H.; Aach, T.; Tischendorf, J.J. Computer-based classification of small colorectal polyps by using narrow-band imaging with optical magnification. *Gastrointestinal endoscopy* **2011**, *74*, 1354-1359, doi:10.1016/j.gie.2011.08.001.
7. Takemura, Y.; Yoshida, S.; Tanaka, S.; Kawase, R.; Onji, K.; Oka, S.; Tamaki, T.; Raytchev, B.; Kaneda, K.; Yoshihara, M.; et al. Computer-aided system for predicting the histology of colorectal tumors by using narrow-band imaging magnifying colonoscopy (with video). *Gastrointestinal endoscopy* **2012**, *75*, 179-185, doi:10.1016/j.gie.2011.08.051.
8. Aihara, H.; Saito, S.; Inomata, H.; Ide, D.; Tamai, N.; Ohya, T.R.; Kato, T.; Amitani, S.; Tajiri, H. Computer-aided diagnosis of neoplastic colorectal lesions using 'real-time' numerical color analysis during autofluorescence endoscopy. *European journal of gastroenterology & hepatology* **2013**, *25*, 488-494, doi:10.1097/MEG.0b013e32835c6d9a.
9. Mori, Y.; Kudo, S.E.; Wakamura, K.; Misawa, M.; Ogawa, Y.; Kutsukawa, M.; Kudo, T.; Hayashi, T.; Miyachi, H.; Ishida, F.; et al. Novel computer-aided diagnostic system for colorectal lesions by using endocytoscopy (with videos). *Gastrointestinal endoscopy* **2015**, *81*, 621-629, doi:10.1016/j.gie.2014.09.008.
10. Mori, Y.; Kudo, S.E.; Chiu, P.W.; Singh, R.; Misawa, M.; Wakamura, K.; Kudo, T.; Hayashi, T.; Katagiri, A.; Miyachi, H.; et al. Impact of an automated system for endocytoscopic diagnosis of small colorectal lesions: an international web-based study. *Endoscopy* **2016**, *48*, 1110-1118, doi:10.1055/s-0042-113609.
11. Kominami, Y.; Yoshida, S.; Tanaka, S.; Sanomura, Y.; Hirakawa, T.; Raytchev, B.; Tamaki, T.; Koide, T.; Kaneda, K.; Chayama, K. Computer-aided diagnosis of colorectal polyp histology by using a real-time image recognition system and narrow-band imaging magnifying colonoscopy. *Gastrointestinal endoscopy* **2016**, *83*, 643-649, doi:10.1016/j.gie.2015.08.004.
12. Misawa, M.; Kudo, S.E.; Mori, Y.; Nakamura, H.; Kataoka, S.; Maeda, Y.; Kudo, T.; Hayashi, T.; Wakamura, K.; Miyachi, H.; et al. Characterization of Colorectal Lesions Using a Computer-Aided Diagnostic System for Narrow-Band Imaging Endocytoscopy. *Gastroenterology* **2016**, *150*, 1531-1532 e1533, doi:10.1053/j.gastro.2016.04.004.
13. Komeda, Y.; Handa, H.; Watanabe, T.; Nomura, T.; Kitahashi, M.; Sakurai, T.; Okamoto, A.; Minami, T.; Kono, M.; Arizumi, T.; et al. Computer-Aided Diagnosis Based on Convolutional Neural Network System for Colorectal Polyp Classification: Preliminary Experience. *Oncology* **2017**, *93 Suppl 1*, 30-34, doi:10.1159/000481227.
14. Chen, P.J.; Lin, M.C.; Lai, M.J.; Lin, J.C.; Lu, H.H.; Tseng, V.S. Accurate Classification of Diminutive Colorectal Polyps Using Computer-Aided Analysis. *Gastroenterology* **2018**, *154*, 568-575, doi:10.1053/j.gastro.2017.10.010.
15. Mori, Y.; Kudo, S.E.; Misawa, M.; Saito, Y.; Ikematsu, H.; Hotta, K.; Ohtsuka, K.; Urushibara, F.; Kataoka, S.; Ogawa, Y.; et al. Real-Time Use of Artificial Intelligence in Identification of Diminutive Polyps During Colonoscopy: A Prospective Study. *Annals of internal medicine* **2018**, *169*, 357-366, doi:10.7326/M18-0249.
16. Byrne, M.F.; Chapados, N.; Soudan, F.; Oertel, C.; Linares Perez, M.; Kelly, R.; Iqbal, N.; Chandelier, F.; Rex, D.K. Real-time differentiation of adenomatous and hyperplastic diminutive colorectal polyps during analysis of unaltered videos of standard colonoscopy using a deep learning model. *Gut* **2019**, *68*, 94-100, doi:10.1136/gutjnl-2017-314547.
17. Sanchez-Montes, C.; Sanchez, F.J.; Bernal, J.; Cordova, H.; Lopez-Ceron, M.; Cuatrecasas, M.; Rodriguez de Miguel, C.; Garcia-Rodriguez, A.; Garces-Duran, R.; Pellise, M.; et al. Computer-aided prediction of polyp histology on white light colonoscopy using surface pattern analysis. *Endoscopy* **2019**, *51*, 261-265, doi:10.1055/a-0732-5250.

18. Jin, E.H.; Lee, D.; Bae, J.H.; Kang, H.Y.; Kwak, M.S.; Seo, J.Y.; Yang, J.I.; Yang, S.Y.; Lim, S.H.; Yim, J.Y.; et al. Improved Accuracy in Optical Diagnosis of Colorectal Polyps Using Convolutional Neural Networks with Visual Explanations. *Gastroenterology* **2020**, *158*, 2169-2179 e2168, doi:10.1053/j.gastro.2020.02.036.
19. Song, E.M.; Park, B.; Ha, C.A.; Hwang, S.W.; Park, S.H.; Yang, D.H.; Ye, B.D.; Myung, S.J.; Yang, S.K.; Kim, N.; et al. Endoscopic diagnosis and treatment planning for colorectal polyps using a deep-learning model. *Scientific reports* **2020**, *10*, 30, doi:10.1038/s41598-019-56697-0.
20. Kudo, S.E.; Misawa, M.; Mori, Y.; Hotta, K.; Ohtsuka, K.; Ikematsu, H.; Saito, Y.; Takeda, K.; Nakamura, H.; Ichimasa, K.; et al. Artificial Intelligence-assisted System Improves Endoscopic Identification of Colorectal Neoplasms. *Clinical gastroenterology and hepatology : the official clinical practice journal of the American Gastroenterological Association* **2020**, *18*, 1874-1881 e1872, doi:10.1016/j.cgh.2019.09.009.
21. Zachariah, R.; Samarasena, J.; Luba, D.; Duh, E.; Dao, T.; Requa, J.; Ninh, A.; Karnes, W. Prediction of Polyp Pathology Using Convolutional Neural Networks Achieves "Resect and Discard" Thresholds. *The American journal of gastroenterology* **2020**, *115*, 138-144, doi:10.14309/ajg.0000000000000429.
22. Ozawa, T.; Ishihara, S.; Fujishiro, M.; Kumagai, Y.; Shichijo, S.; Tada, T. Automated endoscopic detection and classification of colorectal polyps using convolutional neural networks. *Therapeutic advances in gastroenterology* **2020**, *13*, 1756284820910659, doi:10.1177/1756284820910659.
23. Zorron Cheng Tao Pu, L.; Maicas, G.; Tian, Y.; Yamamura, T.; Nakamura, M.; Suzuki, H.; Singh, G.; Rana, K.; Hirooka, Y.; Burt, A.D.; et al. Computer-aided diagnosis for characterization of colorectal lesions: comprehensive software that includes differentiation of serrated lesions. *Gastrointestinal endoscopy* **2020**, *92*, 891-899, doi:10.1016/j.gie.2020.02.042.
24. Zhou, G.; Xiao, X.; Tu, M.; Liu, P.; Yang, D.; Liu, X.; Zhang, R.; Li, L.; Lei, S.; Wang, H.; et al. Computer aided detection for laterally spreading tumors and sessile serrated adenomas during colonoscopy. *PloS one* **2020**, *15*, e0231880, doi:10.1371/journal.pone.0231880.
25. van der Zander, Q.E.W.; Schreuder, R.M.; Fonolla, R.; Scheeve, T.; van der Sommen, F.; Winkens, B.; Aepli, P.; Hayee, B.; Pischel, A.B.; Stefanovic, M.; et al. Optical diagnosis of colorectal polyp images using a newly developed computer-aided diagnosis system (CADx) compared with intuitive optical diagnosis. *Endoscopy* **2021**, *53*, 1219-1226, doi:10.1055/a-1343-1597.
26. Weigt, J.; Repici, A.; Antonelli, G.; Afifi, A.; Kliegis, L.; Correale, L.; Hassan, C.; Neumann, H. Performance of a new integrated computer-assisted system (CADe/CADx) for detection and characterization of colorectal neoplasia. *Endoscopy* **2022**, *54*, 180-184, doi:10.1055/a-1372-0419.
27. Hassan, C.; Balsamo, G.; Lorenzetti, R.; Zullo, A.; Antonelli, G. Artificial Intelligence Allows Leaving-In-Situ Colorectal Polyps. *Clinical gastroenterology and hepatology : the official clinical practice journal of the American Gastroenterological Association* **2022**, *20*, 2505-2513 e2504, doi:10.1016/j.cgh.2022.04.045.
28. Hossain, E.; Abdelrahim, M.; Tanasescu, A.; Yamada, M.; Kondo, H.; Yamada, S.; Hamamoto, R.; Marugame, A.; Saito, Y.; Bhandari, P. Performance of a novel computer-aided diagnosis system in the characterization of colorectal polyps, and its role in meeting Preservation and Incorporation of Valuable Endoscopic Innovations standards set by the American Society of Gastrointestinal Endoscopy. *DEN open* **2023**, *3*, e178, doi:10.1002/deo2.178.
29. Rondonotti, E.; Hassan, C.; Tamanini, G.; Antonelli, G.; Andrisani, G.; Leonetti, G.; Paggi, S.; Amato, A.; Scardino, G.; Di Paolo, D.; et al. Artificial intelligence-assisted optical diagnosis for the resect-and-discard strategy in clinical practice: the Artificial intelligence BLI Characterization (ABC) study. *Endoscopy* **2023**, *55*, 14-22, doi:10.1055/a-1852-0330.
30. Minami, S.; Saso, K.; Miyoshi, N.; Fujino, S.; Kato, S.; Sekido, Y.; Hata, T.; Ogino, T.; Takahashi, H.; Uemura, M.; et al. Diagnosis of Depth of Submucosal Invasion in Colorectal Cancer with AI Using Deep Learning. *Cancers (Basel)* **2022**, *14*, doi: 10.3390/cancers14215361.
31. Lui, T.K.L.; Wong, K.K.Y.; Mak, L.L.Y.; Ko, M.K.L.; Tsao, S.K.K.; Leung, W.K. Endoscopic prediction of deeply submucosal invasive carcinoma with use of artificial intelligence. *Endosc Int Open* **2019**, *7*, E514-E520, doi:10.1055/a-0849-9548.
32. Luo, Y.; Zhang, Y.; Liu, M.; Lai, Y.; Liu, P.; Wang, Z.; Xing, T.; Huang, Y.; Li, Y.; Li, A.; et al. Artificial Intelligence-Assisted Colonoscopy for Detection of Colon Polyps: a Prospective, Randomized Cohort Study. *J Gastrointest Surg* **2021**, *25*, 2011-2018, doi:10.1007/s11605-020-04802-4
33. Tokunaga, M.; Matsumura, T.; Nankinzan, R.; Suzuki, T.; Oura, H.; Kaneko, T.; Fujie, M.; Hirai, S.; Saiki, R.; Akizue, N.; et al. Computer-aided diagnosis system using only white-light endoscopy for the prediction of invasion depth in colorectal cancer. *Gastrointestinal endoscopy* **2021**, *93*, 647-653, doi: 10.1016/j.gie.2020.07.053.
34. Yao, L.; Lu, Z.; Yang, G.; Zhou, W.; Xu, Y.; Guo, M.; Huang, X.; He, C.; Zhou, R.; Deng, Y.; et al. Development and validation of an artificial intelligence-based system for predicting colorectal cancer invasion depth using multi-modal data. *Digestive endoscopy : official journal of the Japan Gastroenterological Endoscopy Society* **2022**, doi:10.1111/den.14493.
